# Four Mixed-Ligand Zn(II) Three-Dimensional Metal-Organic Frameworks: Synthesis, Structural Diversity, and Photoluminescent Property

**DOI:** 10.3390/polym9120644

**Published:** 2017-11-25

**Authors:** Chih-Chieh Wang, Szu-Yu Ke, Chia-Wen Cheng, Yu-Wen Wang, Hsiao-Shan Chiu, Yu-Chien Ko, Ning-Kuei Sun, Mei-Lin Ho, Chung-Kai Chang, Yu-Chun Chuang, Gene-Hsiang Lee

**Affiliations:** 1 Department of Chemistry, Soochow University, Taipei 11102, Taiwan; sarah6017@yahoo.com.tw (S.-Y.K.); wendythu07@gmail.com (C.-W.C.); 01133046@scu.edu.tw (Y.-W.W.); anhhhh@hotmail.com (H.-S.C.); perfectshow15@gmail.com (Y.-C.K.); coco830824@gmail.com (N.-K.S.); 2 National Synchrotron Radiation Research Center, Hsinchu 30076, Taiwan; haha5ckai@gmail.com (C.-K.C.); chuang.yc@nsrrc.org.tw (Y.-C.C.); 3 Instrumentation Center, National Taiwan University, Taipei 10617, Taiwan; ghlee@ntu.edu.tw

**Keywords:** coordination polymer, metal-organic framework, structural topology, interpenetration, photoluminescence

## Abstract

Assemblies of four three-dimensional (3D) mixed-ligand coordination polymers (CPs) having formulas, {[Zn_2_(bdc)_2_(4-bpdh)]·C_2_H_5_OH·2H_2_O}_n_ (**1**), [Zn(bdc)(4-bpdh)]_n_ (**2**), {[Zn_2_(bdc)_2_(4-bpdh)_2_]·(4-bpdh)}_n_ (**3**), and {[Zn(bdc)(4-bpdh)]·C_2_H_5_OH}_n_ (**4**) (bdc^2−^ = dianion of 1,4-benzenedicarboxylic acid, 4-bpdh = 2,5-bis(4-pyridyl)-3,4-diaza-2,4-hexadiene) have been synthesized and structurally characterized by single-crystal X-ray diffraction method. Structural determination reveals that the coordination numbers (geometry) of Zn(II) ions in **1**, **2**, **3**, and **4** are five (distorted square-pyramidal (SP)), six (distorted octahedral (O*_h_*)), five (trigonal-bipyramidal (TBP)), and four (tetrahedral (T*_d_*)), respectively, and are bridged by 4-bpdh with *bis*-monodentate coordination mode and bdc^2−^ ligands with *bis*-bidentate in **1**, chelating/bidentate in **2**, *bis*-monodentate and *bis*-bidentate in **3**, and *bis*-monodentate in **4**, to generate two-fold interpenetrating 3D cube-like metal-organic framework (MOF) with **pcu** topology, non-interpenetrating 3D MOF, two-fold interpenetrating 3D rectangular-box-like MOF with **pcu** topology and five-fold interpenetrating diamondoid-like MOF with **dia** topology, respectively. These different intriguing architectures indicate that the coordination numbers and geometries of Zn(II) ions, coordination modes of bdc^2−^ ligand, and guest molecules play important roles in the construction of MOFs and the formation of the structural topologies and interpenetrations. Thermal stabilities, and photoluminescence study of **1**–**4** were also studied in detail. The complexes exhibit ligands based photoluminescence properties at room temperature.

## 1. Introduction

Coordination polymers (CPs) [1,2], or metal-organic frameworks (MOFs) [1,2], containing guest molecules are very attractive research fields owing to their designable structure, unusual flexibilities, and tunable functional application [3,4,5,6,7,8], which provide a unique opportunity for developing advanced functional materials for studying fundamental nanomolecular assemblies in confined spaces [9,10]. In the past decade, the design and preparation of coordination polymers with MOFs or well-regulated networks have attracted great interest for the sake of the potential applications in catalysis, gas adsorption, nonlinear optics, conductivity, luminescence, and magnetism [11,12,13,14,15,16,17,18]. In principle, the assembly of CPs can be influenced by several factors, such as the reaction condition, nature of anions, coordination geometry of the metal ions, flexibility and coordination modes of the ligands, temperature, solvent, and so on [19,20]. Among these factors, the coordination geometry of the central metal ions and flexibility and coordination modes of the ligands have been well demonstrated that they can control the structural topology, pore size, and interpenetration of MOFs, resulting in the structural diversity and related properties [13,14,15,21,22,23]. Based on this concept, utilizing suitable organic spacers with functional groups that are capable of bridging metal centers to construct the well-defined crystalline materials has shown to be a fast developing research in connection with crystal engineering, coordination chemistry, supramolecular chemistry, and material science [24,25,26,27,28]. A framework with long spacer ligands can be interwoven by the incorporation of other nets, resulting in the formation of interpenetrating architectures [29,30,31,32,33]. The degree of interpenetration relies on factors that include temperature [34], concentration, templates [35], ligand type [36], and guest removal/inclusion [37,38,39,40]. For this purpose, constructing a series of three-dimensional (3D) MOFs with different structural topology under the same synthetic condition and discussing the variable factors, including the coordination geometry, and coordination modes, and structural conformation of ligands, has become an interesting issue. Recently, diaza-base ligands, such as 1,4-bis(4-pyridyl)-2,3-diaza-1,3-butadiene (4-bpd), 2,5-bis(4-pyridyl)-3,4-diaza-2,4-hexadiene (4-bpdh), 1,4-bis(3-pyridyl)-2,3-diaza-1,3-butadiene (3-bpd), and 2,5-bis(3-pyridyl)-3,4-diaza-2,4-hexadiene (3-bpdh) with the zigzag conformation of the spacer diaza moiety (–CR=N–N=CR–) between the two pyridyl groups have been justified to play important roles in the design of the multi-dimensional MOFs [41,42,43,44,45,46,47,48,49,50,51,52,53,54,55,56,57,58,59,60,61,62,63,64,65,66,67,68,69,70,71,72]. Many three-dimensional (3D) structural topologies have been constructed either by using various metal centers bridged by one of the diaza-base ligands that is associated with one multi-carboxylate ligands, or by using one metal center bridged by one of the diaza-base ligand associated with various multi-carboxylate ligands [52,53,54,55,56,57,58,59,60,61,62,63,64,65,66,67,68,69,70,71,72]. However, the structural diversity of 3D CPs constructed based only on one metal center bridged by the same diaza-base ligand and dicarboxylate ligands under the same synthetic condition is rare and worth systematic study. Focusing on this approach, we report here four 3D Zn(II)-bpdh-bdc CPs, {[Zn_2_(bdc)_2_(4-bpdh)]·C_2_H_5_OH·2H_2_O}_n_ (**1**), [Zn(bdc)(4-bpdh)]_n_ (**2**), {[Zn_2_(bdc)_2_(4-bpdh)_2_]·(4-bpdh)}_n_ (**3**), and {[Zn(bdc)(4-bpdh)]·C_2_H_5_OH}_n_ (**4**) (where 4-bpdh = 2,5-bis(4-pyridyl)-3,4-diaza-2,4-hexadiene) and bdc^2−^ = dianion of 1,4-benzenedicarboxylic acid), in which the Zn(II) centres adopts four different kinds of coordination geometries bridged by the 4-bpdh with a *bis*-monodentate coordination modes and bdc^2−^ ligands with three kinds of coordination modes, including *bis*-bidentate, chelating/bidentate, and *bis*-monodentate, to construct their 3D MOFs with different structural topology and network interpenetration. 

## 2. Materials and Methods 

### 2.1. Materials and Physical Techniques

General: 4-bpdh was synthesized according to the literature procedure [41]. All of the chemicals were of reagent grade and were used as commercially obtained without further purification. Elementary analyses (carbon, hydrogen and nitrogen) were performed using a Perkin-Elmer 2400 elemental analyzer (PerkinElmer, Taipei, Taiwan). IR spectra were recorded on a Nicolet Fourier Transform IR MAGNA-IR 500 spectrometer (Thermo Fisher Scientific, Waltham, MA, USA) in the range of 500–4000 cm^−1^ using the KBr disc technique. Thermogravimetric analysis (TGA) of compounds **1**–**4** was performed on a computer-controlled Perkin-Elmer 7 Series/UNIX TGA7 analyzer (PerkinElmer, Taipei, Taiwan). Single-phased powder samples were loaded into alumina pans and heated with a ramp rate of 5 °C/min from room temperature to 700 °C under a nitrogen atmosphere. The adsorption isotherm of N_2_, H_2_ (77 K), and CO_2_ (195 K) for **1**–**4** was measured in the gaseous state by using BELSORP-max volumetric adsorption equipment from BEL, Osaka, Japan. In the sample cell (~1.8 cm^3^), maintained at T ±0.03 K, was placed the adsorbent sample (~100–150 mg), which has been prepared at 180 °C for **1**–**4** and 10^−2^ Pa for about 24 h prior to measurement of the isotherm. The adsorbate was placed into the sample cell, and then the change of pressure was monitored and the degree of adsorption was determined by the decrease of pressure at equilibrium state. All of the operations were through automatically computer-controlled.

### 2.2. Synthesis of {[Zn_2_(bdc)_2_(4-bpdh)]·C_2_H_5_OH·2H_2_O}_n_ *(**1**)* and [Zn(bdc)(4-bpdh)]_n_ *(**2**)*

An ethanol/H_2_O solution (1:1, 4 mL) of terephthalic disodium salt (Na_2_bdc 0.025 mmol) was added to an ethanol/water (1:1, 8 mL) solution of ZnCl_2_ (0.025 mmol) and 2,5-bis(4-pyridyl)-3,4-diaza-2,4-hexadiene (0.050 mmol) at room temperature. After standing for one week, light-yellow block crystals of 1 (yield, 35.3% based on terephthalic disodium salt) and light-yellow plate crystals of 2 (yield, 24.9% based on terephthalic disodium salt) were obtained, which are suitable for X-ray diffraction analysis. The resulting crystals of **1** and **2** were collected by filtration, washed several times with distilled water, and dried in air. Anal. Calc. for de-solvated (**1**), C_30_H_14_N_4_O_8_Zn_2_: C 52.28, N 8.13, H 2.04. Found: C 51.85, N 8.01, H 2.54. IR (KBr pellet): ν = 3400 (m), 1638 (s), 1616 (vs), 1504 (m), 1426 (m), 1390 (s), 1298 (m), 1064 (w), 1029 (w), 1016 (w), 887 (w), 825 (m), 749 (s) cm^−1^. Anal. Calc. for C_22_H_18_N_4_O_4_Zn_1_ (**2**): C 56.49, N 11.98, H 3.88; Found: C 56.29, N 11.86, H 3.84. IR (KBr pellet): ν = 1608 (s), 1572 (vs), 1503 (s), 1390 (vs), 1331 (m), 1303 (m), 1223 (m), 1066 (m), 1016 (m), 835 (s), 754 (s) cm^−1^.

### 2.3. Synthesis of [Zn(bdc)(4-bpdh)]_n_ *(**2**)*, {[Zn_2_(bdc)_2_(4-bpdh)_2_]·(4-bpdh)}_n_ *(**3**)* and {[Zn(bdc)(4-bpdh)]·C_2_H_5_OH}_n_ *(**4**)*

An ethanol/H_2_O (1:3) solution (4 mL) of terephthalic disodium salt (Na_2_bdc 0.025 mmol) was added to an ethanol/water (1:3) solution (8 mL) of ZnCl_2_ (0.025 mmol) and 2,5-bis(4-pyridyl)-3,4-diaza-2,4-hexadiene (4-bpdh) (0.05 mmol) at room temperature. After standing for one week, the light-yellow plate crystals of **2** (yield, 22.8% based on terephthalic disodium salt), yellow block crystals of **3** (yield, 12.2% based on terephthalic disodium salt), and orange-yellow block crystals of **4** (yield, 30.2% based on terephthalic disodium salt), respectively, were obtained, which are suitable for X-ray diffraction analysis. The resulting crystals of **2**–**4** were collected by filtration, washed several times with distilled water, and dried in air. Anal. Calc. for C_22_H_18_N_4_O_4_Zn_1_ (**2**): C 56.49, N 11.98, H 3.88; Found: C 56.29, N 11.86, H 3.84. IR (KBr pellet): ν = 1608 (s), 1572 (vs), 1503 (s), 1390 (vs), 1331 (m), 1303 (m), 1223 (m), 1066 (m), 1016 (m), 835 (s), 754 (s) cm^−1^. Anal. Calc. for desolvated (**3**), C_44_H_36_N_8_O_8_Zn_2_: C 56.49, N 11.98, H 3.88; Found: C 56.27, N 12.08, H 4.15. IR (KBr pellet): ν = 1615 (vs), 1570 (s), 1500 (m), 1397 (s), 1367 (s), 1292 (m), 1220 (m), 1091 (m), 1065 (w), 1019 (w), 830 (m), 753 (m) cm^−1^. Anal. Calc. for C_24_H_24_N_4_O_5_Zn_1_ (**4**): C 56.10, N 10.91, H 4.71; Found: C 56.68, N 11.18, H 4.62. IR (KBr pellet): ν = 3384 (m), 1616 (vs), 1568 (s), 1500 (m), 1403 (s), 1344 (s), 1292 (m),1220 (m), 1140 (m), 1089 (w), 1064 (m), 1027 (m), 884 (w), 830 (m), 745 (m). 

### 2.4. Crystallographic Data Collection and Refinements

Single-crystal structure analysis for compounds **1**–**4** was performed out on a Siemens SMART diffractomer with a charge coupled detector (CCD) detector with Mo radiation (λ = 0.71073 Å) at 100 K. A preliminary orientation matrix and unit cell parameters were determined from 3 runs of 15 frames each, each frame correspond to a 0.3° scan in 10 s, following by spot integration and least-squares refinement. For each structure, data were measured using ω scans of 0.3° per frame for 20 s until a complete hemisphere had been collected. Cell parameters were retrieved using SMART [73] software and refined with SAINT [74] on all of the observed reflections. Data reduction was performed with the SAINT [75] software and corrected for Lorentz and polarization effects. Absorption corrections were applied with the program SADABS [75]. Direct phase determination and subsequent difference Fourier map synthesis yielded the positions of all the atoms, which were subjected to anisotropic refinements for non-hydrogen atoms and isotropic for hydrogen atoms. The final full-matrix, least-squares refinement on *F*^2^ was applied for all of the observed reflections (I > 2σ(I)). All of the calculations were performed by using the SHELXTL-PC V 5.03 software package [76]. Crystal data and details of the data collection and structure refinements for 1–4 are summarized in Table 1. CCDC-1478481, 1478482, 1478483 and 1478484 for **1**, **2**, **4**, and **3**, respectively, contains the supplementary crystallographic data for this paper. These data can be obtained free of charge at www.ccdc.cam.ac.uk/conts/retrieving.html or from the Cambridge Crystallographic Data Centre, 12, Union Road, Cambridge CB2 1EZ, UK; fax: (internat.) +44-1223/336-033; email: deposit@ccdc.cam.ac.uk.

### 2.5. In Situ X-ray Powder Diffraction

The powder X-ray diffraction patterns of compounds **1**–**4** was measured at the BL01C2 beamline of National Synchrotron Radiation Research Center (NSRRC) in city, Taiwan. The wavelength of the incident X-rays was 1.0332 Ǻ (12.0 KeV), delivered from the superconducting wavelength-shifting magnet and a Si(111) double-crystal monochromator. The diffraction pattern was recorded with a Mar345 imaging-plate detector and typical exposure duration of 2 min. The one-dimensional powder diffraction profile was converted with GSAS-II program [77]. The diffraction angles were calibrated according to Bragg positions of lanthanum boride (SRM660b) standards.

### 2.6. Spectral Measurement

Compounds **1**–**4** were obtained with a HITACHI U-3900Hspectrophotometer (Hitachi High Technologies America, Inc., Schaumburg, IL, USA) equipped with an integrating sphere accessory (Al_2_O_3_ was used as a reference). In order to investigate the possible differences in photoluminescence of **1**–**4** due to the variation of in topology, **1**–**4** were measured with a confocal mode of a Mono-Vista confocal Raman microscope system (Princeton Instruments, Acton, MA, USA). In this approach, a 325 nm laser line was used as an excitation source throughout the measurements. The photoluminescence was separated from the scattering light of excitation pulse by an edge filter with a cut-off wavelength of 325 nm. Subsequently, the luminescence was collected by an optical assembly to the entrance slit of a polychromator (blazed at 500 nm), which was coupled with a sensitive charge coupled detector (CCD, Princeton Instruments, PI-MAX). The CCD was operated in shutter mode, and the measurements were performed with 100 ms exposure time.

## 3. Results and Discussion

### 3.1. Syntheses and Characterization of Compounds ***1**–**4***

**Scheme 1:**
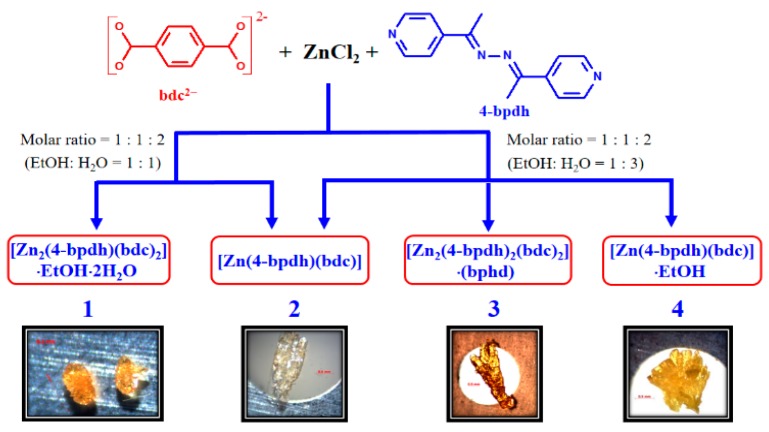
Synthetic representation of compounds **1**–**4**.

Four CPs **1**–**4** with different 3D MOFs were synthesized on the basis of the selection of reaction solvent systems, while the other synthetic parameters were intentionally held constant. The dimensionality and topology of the networks that were produced in this work are determined by the coordination environments of Zn(II) and bridging modes of bdc^2−^ and 4-bpdh ligands, which are clearly dictated by the solvent molecules. The reaction in EtOH/H_2_O (12 mL, *v*/*v* 1:1) solvent results in the formation of **1** and **2**, while **2**–**4** are formed in EtOH/H_2_O (12 mL, *v*/*v* 1:3) solvent (Scheme 1). The experiments reveal that the EtOH/H_2_O ratios are critical to form supramolecular isomers **1**–**4**. In order to explore the role of EtOH/H_2_O ratios on the assembled system of Zn(II)/bdc^2−^/4-bpdh, we carried out a series of related experiments by changing the volumetric ratio of EtOH/H_2_O (12 mL) from 5:1 to 1:5. The volumetric ratio of EtOH/H_2_O used in the synthetic processes and the related yields of **1**–**4** are recorded in a tabular form (Table S1 deposited in the Supplementary Information). However, the formations of the CPs **1**–**4** show specificities to the volumetric ratios of 1:1 and 1:2, respectively, at least for the current experimental results. It clearly shows that the solvent ratios have effects on the final products and the simple change of mixed solvent ratio can generate quite different structures of the resultant CPs. All of the products were confirmed through powder XRD measurement of the as-synthesized samples (Figures S1b–S4b deposited in the Supplementary Information). The powder X-ray diffraction (PXRD) patterns of these compounds are in a good agreement with the simulated ones calculated from the corresponding single-crystal X-ray diffraction data. The most relevant IR features for **1**–**4** are those that are associated with the bdc^2−^ ligands and almost identical throughout the region from 500 to 4000 cm^−1^, showing strong characteristic absorption bands in 1300–1600 cm^−1^ range, attributed to the carboxylate groups of bdc^2−^ ligands.

### 3.2. Structural Description of {[Zn_2_(bdc)_2_(4-bpdh)]·C_2_H_5_OH·2H_2_O}_n_ *(**1**)*

**Scheme 2:**
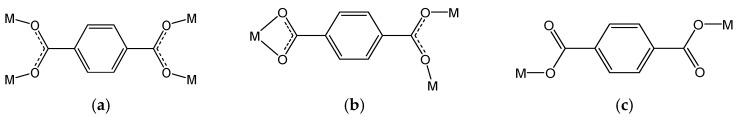
Coordination modes of (**a**) *bis*-bidentate, (**b**) chelating/bidentate, and (**c**) *bis*-monodentate of bdc^2−^ ligand used in compounds **1**–**4**.

**Figure 1:**
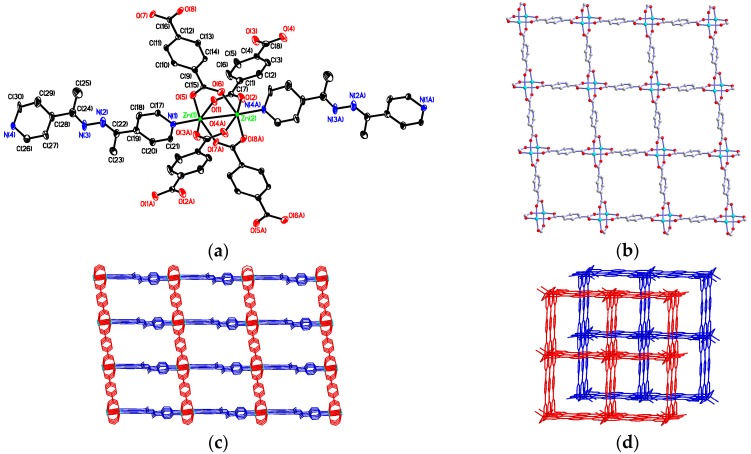
(**a**) Square-pyramidal coordination environments about Zn(II) ion in **1** with atom labelling scheme (Oak Ridge Thermal Ellipsoid Plot (ORTEP) drawing, 30% thermal ellipsoids). The solvent molecules and H atoms are omitted for clarity; (**b**) The two-dimensional (2D) layered framework constructed by Zn(II) and bdc^2−^ ligands with *bis*-bidentate coordination mode; (**c**) The three-dimensional (3D) cube-like metal-organic frameworks (MOF); (**d**) The two-fold interpenetrating 3D supramolecular architecture of **1**.

Compound **1** crystallizes in the triclinic *P*-1 space group, in which the asymmetric unit contains two Zn(II) metal center, two bdc^2−^, one 4-bpdh ligands and one ethanol, two water guest molecules. The molecular structure of **1** shown in Figure 1a reveals that two crystallographically independent Zn(II) ions (Zn(1) and Zn(2)) are both five coordinate bonded with four oxygen atoms of four bdc^2−^ ligands in the equatorial plane with Zn–O distances in the range of 2.010(7)–2.050(7) Å and one nitrogen atom of one 4-bpdh ligands at the axial position with Zn–N distances of 2.012(8) and 2.031(8) Å, forming a distorted square-pyramidal (SP) geometry. The selected bond lengths and angles around the Zn(II) centres are listed in Table S2 (deposited in the Supplementary Information). In **1**, four bdc^2−^ ligands adopting *bis*-bidentate coordination modes (Scheme 2a) connect two Zn(II) centres generating a paddle-wheel dinuclear [Zn_2_(CO_2_)_4_] secondary building unit (SBU), and each dinuclear SBU unit bridged by bdc^2−^ ligands to form two-dimensional (2D) planar layered frameworks with (4,4) topology along the crystallographic *ab* plane (Figure 1b). Adjacent layers are further pillared by 4-bpdh ligands with *bis*-monodentate coordination mode to complete its 3D cube-like open MOF (Figure 1c). Topological analysis by TOPOS [78] suggests the presence of a dinuclear [Zn_2_(CO_2_)_4_] unit, which act as a 6-connecting node, and the overall structure has a cubic (**pcu**) net topology. In each dinuclear SBU unit, two Zn(II) centres are separated by 2.939(2) Å and the Zn(II)···Zn(II) separations are 10.893(2) and 15.211(3) Å through bdc^2−^ and 4-bpdh bridges, respectively. The intra-framework spaces are then occupied by the other crystallographically identical but independent networks, which interpenetrate the first one to form a two-fold interpenetrating 3D supramolecular architecture (as shown in Figure 1d) with the vacant pores intercalated with the ethanol and water molecules. The solvent-accessible volume is 23.7% (calculated with PLATON [79]) after the removal of guest solvent molecules. It is noteworthy that similar 3D MOF was constructed by Zn(II) ion with the same ligands, but display a three-fold interpenetrated coordination network found in the {[Zn(Meazpy)_0.5_(terep)]}*_n_* without any guest solvent molecules (Meazpy = *N*,*N*’-*bis*-(1pyridin-4-ylethylidene), terep = terephthalate) [63].

### 3.3. Structural Description of [Zn(bdc)(4-bpdh)]_n_ *(**2**)*

**Figure 2:**
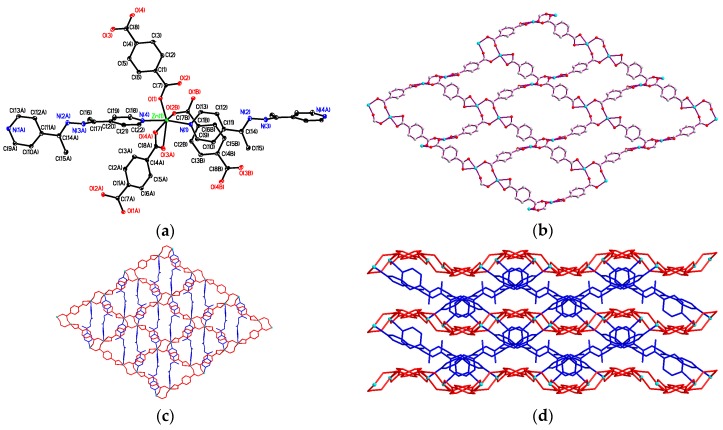
(**a**) Distorted octahedral coordination environments about Zn(II) ion in **2** with atom labelling scheme (ORTEP drawing, 30% thermal ellipsoids). The H atoms are omitted for clarity; (**b**) The 2D sinusoidal-like layered framework constructed by Zn(II) and bdc^2−^ ligands with chelating/bidentate coordination mode viewing along the *bc* plane; (**c**) The 3D MOF constructed by the 2D [Zn–bdc] layers (red) bridged by 4-bpdh ligands (blue); (**d**) The 3D non-interpenetrating coordination network of **2**.

Compound **2** crystallizes in the monoclinic *P* 2_1_/c space group, in which the asymmetric unit contains one Zn(II) metal center, one bdc^2−^, and one 4-bpdh ligands. The ORTEP drawing view with atom numbering scheme of the Zn(II) coordination geometry is shown in Figure 2a, in which the Zn(II) ion is six-coordinate bonded to four oxygen atoms from three bdc^2−^ ligands with Zn–O distances in the range of 2.045(2)–2.354(3) Å and two nitrogen atoms from two 4-bpdh ligands with Zn–N distances of 2.155(2) and 2.226(2) Å to form a distorted octahedral (O*_h_*) geometry. The selected bond lengths and angles around the Zn(II) centres are listed in Table S3 (deposited in the Supplementary Information). In **2**, four bdc^2−^ ligands adopting chelating/bidentate coordination mode (Scheme 2b) connect two Zn(II) centres, generating a [Zn_2_(CO_2_)_4_] unit, and each dinuclear unit is bridged by bdc^2−^ ligand to form 2D sinusoidal-like layered frameworks (Figure 2b) with (4,4) topology along the crystallographic *bc* plane. Adjacent layers are further interlinked via the connectivity between the dinuclear Zn(II) units and 4-bpdh ligands with *bis*-monodentate coordination mode in an alternate cross-linkage manner (Figure 2c) to complete its 3D MOF (Figure 2c,d). Such linkage manner of 4-bpdh ligands shown in **2** prevents the interpenetration of the unique 3D MOF. In each dinuclear unit, two Zn(II) centres are separated by 4.493(1) Å and the Zn(II)···Zn(II) separations are 8.967(1) and 10.859(1) Å through bdc^2−^ bridges and 13.549(1) Å through 4-bpdh bridge, respectively.

### 3.4. Structural Description of {[Zn_2_(bdc)_2_(4-bpdh)_2_]·(4-bpdh)}_n_ *(**3**)*

**Figure 3:**
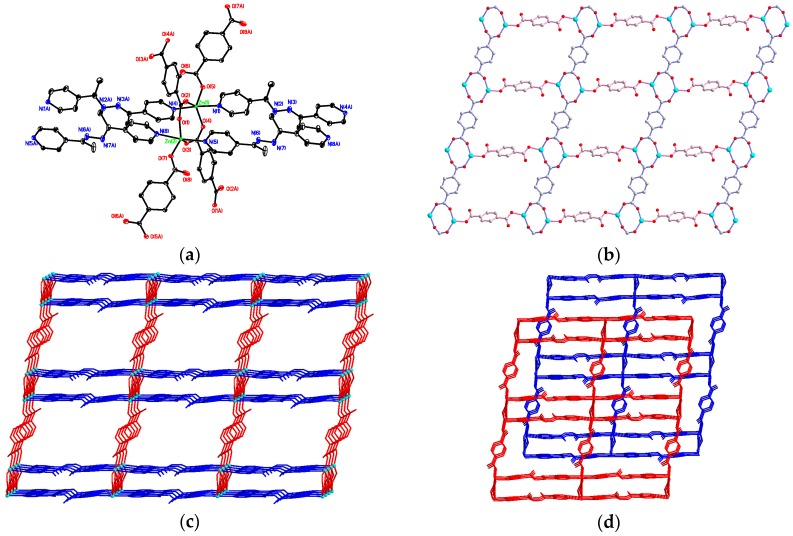
(**a**) Distorted trigonal bipyramidal coordination environments about two Zn(II) ions in **3** with atom labelling scheme (ORTEP drawing, 30% thermal ellipsoids). The guest 4-bpdh molecules and H atoms are omitted for clarity; (**b**) The 2D layered framework constructed by Zn(II) and bdc^2−^ ligands with *bis*-bidentate and *bis*-monodentate coordination modes; (**c**) The 3D rectangular-box-like MOF; (**d**) The two-fold interpenetrating 3D supramolecular architecture of **3**.

Compound **3** crystallizes in the triclinic *P*-1 space group, in which the asymmetric unit contains two Zn(II) metal center, two bdc^2−^, two 4-bpdh ligands, and one free 4-bpdh guest molecule. The molecular structure of **3**, as shown in Figure 3a, reveals that two crystallographically independent Zn(II) ions (Zn(1) and Zn(2)) are both five coordinate bonded with three oxygen atoms of three bdc^2−^ ligands in the equatorial plane with Zn–O distances in the range of 1.964(2)–2.034(2) Å and two nitrogen atoms of two 4-bpdh ligands at the axial positions with Zn–N distances in the range of 2.141(3)–2.228(3) Å forming a distorted trigonal-bipyramidal (TBP) geometry. The selected bond lengths and angles around the Zn(II) centres are listed in Table S4 (deposited in the Supplementary Information). In **3**, four bdc^2−^ ligands adopting two different coordination modes, *bis*-bidentate and *bis*-monodentate (Scheme 2a,c), connect two Zn(II) centres generating a [Zn_2_(CO_2_)_4_] unit, and each dinuclear unit bridged by bdc^2−^ ligands to form 2D planar layered frameworks (Figure 3b), with (4,4) topology along the crystallographic *ab* plane. Adjacent layers are further connected via two crystallographically independent 4-bpdh ligands, with *bis*-monodentate coordination mode to complete its 3D rectangular-box-like open MOF (Figure 3c). Topological analysis by TOPOS [78] suggests the presence of two types of Zn(II) ions that act as 6-connecting nodes, and the overall structure has a cubic (**pcu** α-Po) net topology. In each dinuclear unit, two Zn(II) centres are separated by 3.808(1) Å and the Zn(II)···Zn(II) separations are 10.479(1) and 11.076(1) Å through *bis*-bidentate and *bis*-monodentate bdc^2−^ bridges, respectively, and 15.605(1) Å through 4-bpdh bridge. The intra-framework spaces are then occupied by the other crystallographically identical but independent networks, which interpenetrate the first one to form a two-fold interpenetrating 3D supramolecular architecture (as shown in Figure 3d) with the vacant pores intercalated with guest 4-bpdh molecules. The effective solvent accessible void volume calculated using PLATON [79] is ~16.8% per unit cell volume after the removal of guest 4-bpdh molecules.

### 3.5. Structural Description of {[Zn(bdc)(4-bpdh)]·C_2_H_5_OH}_n_ *(**4**)*

**Figure 4:**
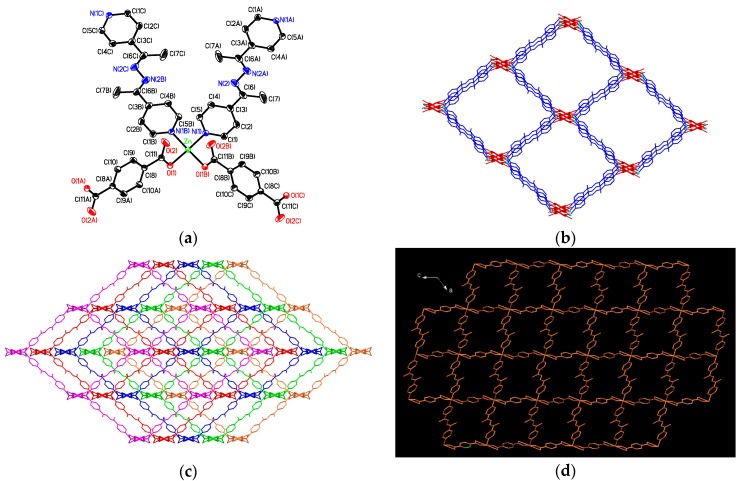
(**a**) Distorted tetrahedral coordination environments about Zn(II) ion in **4** with atom labelling scheme (ORTEP drawing, 30% thermal ellipsoids). The solvent molecules and H atoms are omitted for clarity; (**b**) The 3D diamondoid-like MOF; (**c**) The five-fold interpenetrating 3D supramolecular architecture of **4**; (**d**) The one-dimensional (1D) channels created by the five-fold interpenetrating 3D architecture viewing along the *b* axis.

Compound **4** crystallizes in monoclinic *C*2/c space group, in which the asymmetric unit contains one Zn(II) metal center located at the inversion center, one bdc^2−^, one 4-bpdh ligands, and one EtOH guest molecules. The molecular structure of **4**, shown in Figure 4a, reveals that the Zn(II) ion is four-coordinate bonded with two oxygen atoms of two bdc^2−^ with Zn–O distance of 1.936(2) Å and two nitrogen atoms of two 4-bpdh ligands with Zn–N distance of 2.032(2) Å forming a distorted tetrahedral (T*_d_*) geometry. The selected bond lengths and angles around the Zn(II) centres are listed in Table S5 (deposited in the Supplementary Information). In **4**, the bdc^2−^ and 4-bpdh both acts as bridging ligand with *bis*-monodentate (Scheme 2c) coordination modes connection the Zn(II) ions to form a 3D diamondoid-like MOF (Figure 4b) with **dia** structural topology. The Zn(II)···Zn(II) separations are 10.925(1) and 15.227(8) Å through bdc^2−^ and 4-bpdh bridges, respectively. Topological analysis by TOPOS [78] suggests that each Zn(II) atom acts as a 4-connected node and the overall structure has a (**dia**) net topology. The much larger intra-framework spaces are occupied by four other crystallographically identical but independent networks, which interpenetrate the first one, as well as each other to form a five-fold interpenetrating 3D supramolecular architecture (as shown in Figure 4c), generating one-dimensional (1D) channels along the *b* axis (Figure 4d) intercalated with ethanol molecules. The effective solvent accessible void volume calculated using PLATON [79] is ~27.4% per unit cell volume after the removal of the ethanol molecules.

### 3.6. Thermal Stability of CPs ***1**–**4***

To assess the thermal stability and structural variation as a function of the temperature, thermogravimetric (TG) analysis and in-situ temperature dependent XRD measurements of compounds **1**–**4** were performed on single-phase polycrystalline samples (Figures S1a–S4a deposited in Supplementary Information). During the heating process, the TG analysis (see Figure S1a in the Supplementary Information) revealed that compound **1** underwent a two-step weight loss and the first weight loss of 10.2% (calc. 9.4%), corresponding to the loss of solvated ethanol and water molecules that occurred in the range of approximate 29–71 °C, and then thermal stable up to 234 °C without any weight loss. On further heating, these samples decomposed at approximately 234–500 °C. The TG analysis of **2** and **3** (Figures S2a and S3a in the Supplementary Information) revealed that **2** and **3** are thermally stable up to 310 and 217 °C, respectively, and then underwent a one-step but continuous weight loss in the temperature range of 310–500 °C for **2** and 217–480 °C for **3**, respectively. The TG analysis of **4** (Figure S4a in the Supplementary Information) shows that the first weight loss of 7.6% (calc. 8.2%), corresponding to the loss of ethanol molecules, occurred in the range of approximate 40–100 °C, and then thermal stable up to 235 °C. On further heating, these samples decomposed at approximately 235–500 °C. All of the final products of compounds **1**–**4** is probably zinc oxide (ZnO). To gain the structural changes in depth, in situ synchrotron X-ray powder diffraction patterns of **1** were collected continuously from 25 °C to 410 °C, and the results at some specific temperatures are shown in Figure S1b (deposited in the Supplementary Information). Based on the result of TG analysis, the solvated solvent molecules in the channels of **1** are lost even at RT. The powder XRD patterns of **1** at RT, 50 °C and 80 °C reveal that they are a little different to the simulation one of **1**. This result may be due to the continuous changing in the composition of solvated solvents. However, it shows a phase transition at 90 °C, and then stable in the temperature range from 110 °C to 230 °C. As the temperature rising above to 260 °C to 350 °C, **1** transforms to another crystalline phase and finally decomposed at above 450 °C. The powder XRD pattern (Figure S2b deposited in the Supplementary Information) of **2** at RT is almost identical to the simulation one obtained from single-crystal X-ray diffraction data. There is no phase transition below 290 °C based on the powder XRD patterns shown in Figure S2b. At about 290 °C, the phase transition occurred and further phase transition at 320 °C was observed. As the temperature rising to above 380 °C, **2** decomposed and transformed to metal oxide. The powder XRD pattern (Figure S3b deposited in the Supplementary Information) of **3** at RT showed no obvious different to that at RT with solvents and also similar to the simulation one obtained from single-crystal X-ray diffraction data. This result indicates that the solvated solvents in **3** are relatively strong intermolecular interaction with the main framework. The powder XRD pattern at 140 °C of dry sample showed an obvious structural change of the two-fold interpenetration frameworks due to the loss of solvents. At above 200 °C, the second phase transition occurred and at above 290 °C, the XRD pattern changed and gradually transform to simple metal oxide at above 320 °C. The powder XRD pattern (Figure S4b deposited in the Supplementary Information) of **4** at RT is almost identical to the simulation one that was obtained from single-crystal X-ray diffraction data. There is no phase transition below 80 °C based on the powder XRD patterns shown in Figure S4b. At about 110 °C, the phase transition occurred and further phase transition at 230 °C was observed. As the temperature rising to above 350 °C, **4** decomposed and transformed to metal oxide. All of the in-situ PXRD measurements are in consistent with the TG analyses.

### 3.7. Factors Affecting the Structural Diversity and Interpenetrating of CPs ***1**–**4***

As discussed above, it is interesting to note that, under the same synthetic condition (the same starting materials of Zn(II) salt and mixed ligands, bdc^2−^ and 4-bpdh), the four CPs exhibit different 3D framework structure, and compounds **2**, **3**, and **4** can be described as supramolecular isomers [80]. The structural diversity among **1**–**4** are mainly attributed to the different building blocks, and the various coordination modes of bdc^2−^ ligands and conformations of 4-bpdh ligand, which play important roles on the construction of their 3D frameworks, and structural interpenetration. First of all, different coordination numbers and geometries of Zn(II) centres are undoubtedly an important factor influencing the structural topology of **1**–**4**, in this study, the coordination geometry (coordination number (C. N.)) of Zn(II) centres are SP (5), distorted O*_h_* (6), TBP (5), and T*_d_* (4) for **1**, **2**, **3**, and **4**, respectively. The second important factor is the different coordination mode of bdc^2−^ ligands used in CPs **1**–**4**, including *bis*-bidentate coordination mode in **1**, chelating/bidentate coordination mode in **2**, hybrid *bis*-bidentate and *bis*-monodentate coordination modes in **3**, and *bis*-monodentate coordination mode in **4**, respectively. Different coordination geometries of Zn(II) centres associated with 4-bpdh and bdc^2−^ with different coordination modes not only generate different 3D coordination frameworks, but also create different vacant spaces for the structural interpenetration. For **1** and **3**, the smaller void spaces constructed by the cube-like and rectangular-box-like 3D MOFs intercalated with guest molecules (EtOH and H_2_O for **1** and 4-bpdh for **3**) results in the formation of two-fold interpenetrating supramolecular architecture. However, for **4**, the diamondoid 3D MOF with larger void space results in the formation of a five-fold interpenetrating supramolecular architecture. Many similar 3D diamondoid frameworks constructed by Schiff-base ligands (3-, 4-bpd and 3-, 4-bphd) and muli-carboxylate ligands all display highly (5-fold, 6-fold, and 7-fold) interpenetrating architectures [52,53,54,55,56,57,58,59,60,61,62,63,64,65,66,67,68,69,70,71,72]. In **2**, the alternate cross-linkage bridging manner and the steric hindrance of two methyl groups in 4-bpdh ligands prevents the mutually interpenetration of the unique 3D MOFs, and shows a non-interpenetrating coordination network. These results described above may be concluded that the coordination numbers and geometries of metal centres, the coordination modes of bdc^2−^ ligand, and guest molecules all play an important key factor on the influence of the structural interpenetration.

### 3.8. Gas-Sorption Studies of CPs ***1**–**4***

The thermal stability of microporous MOF associated with gases de-/ad-sorption properties may play a key role for the potential application on the gas storage. To examine the porous nature of compounds **1**–**4**, we have performed gas adsorption studies with N_2_, H_2_ at 77 K, and CO_2_ at 195 K, respectively. In the cases of compounds **1**, **3**, and **4**, although they contain lattice water, ethanol solvents (**1** and **4**), or free 4-bphd ligand (**3**), the pores are blocked due to the two-fold (**1** and **3**) and five-fold (**4**) interpenetration of the 3D networks. As a result, they show negligible or much less uptake N_2_, H_2_, and CO_2_ adsorption (Figure S5 deposited in the Supplementary Information), indicating the blockage of pores. In compound **2**, the non-interpenetrating 3D network is constructed by 2D [Zn(bdc)] layered frameworks, bridged by 4-bphd ligands in a cross-linkage manner, which prevents the entrance of solvent molecule. As a result, it also shows negligible N_2_, H_2_, and CO_2_ adsorption, indicating the blockage of pores.

### 3.9. Luminescence Property of CPs ***1**–**4***

**Figure 5:**
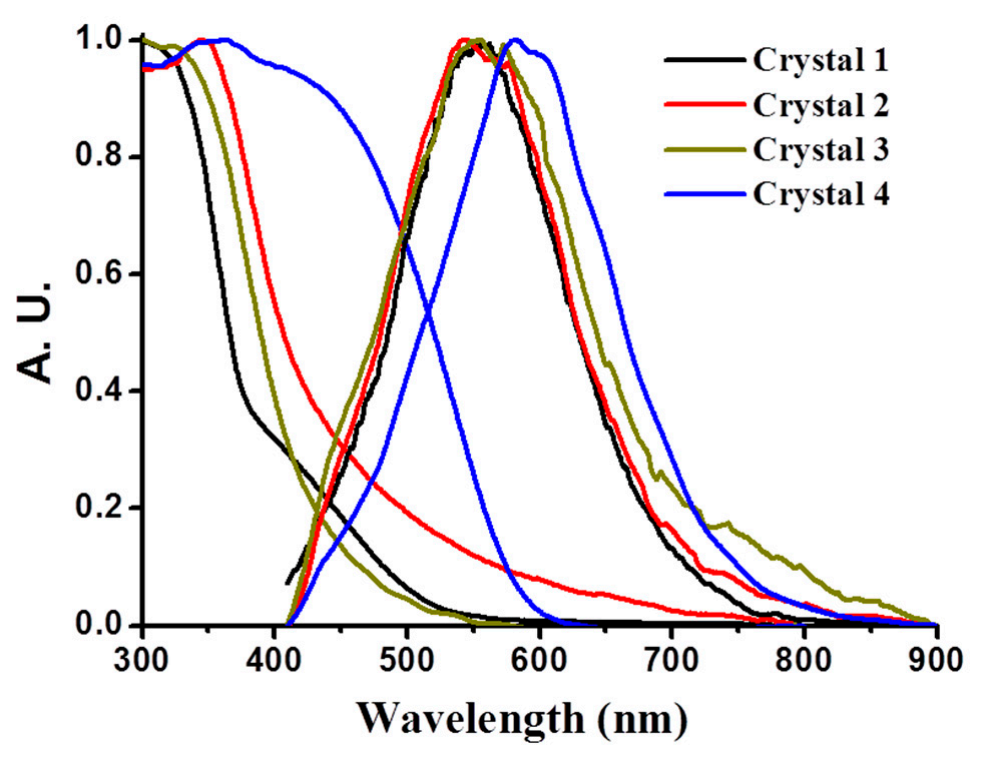
The UV–vis diffusive reflectance and emission spectra of **1**–**4** at λ_ex_ = 325 nm.

The solid-state adsorption and emission properties of **1**–**4** were also investigated at room temperature. The title compounds all have strong absorption at <350 nm, a 325 nm laser line was therefore used as an excitation source throughout the measurements. As shown in Figure 5, the adsorption and emission bands of **1**–**4** in single crystal form are broad and structureless. Upon 325 nm excitation, the emission spectra with maxima centered around 550 nm for **1**–**3**, and 592 nm for **4** were observed, respectively. From our previous study [81], we know that the free ligand Na_2_bdc in single crystal form exhibits a fluorescence with a peak wavelength at 419 nm, which is attributed to the π*–n transitions. The emission band of 4-bpdh in the powder form with center around 570 nm and assigned it to the π*–n transitions (Figure S6 in the Supplementary Information). Also, according the report of Nagaraja et al., the emissions from compound [Zn_4_(muco)_4_(4bpdh)_4_].4bpdh.2H_2_O (where, muco = trans, trans-muconate dianion) might be arising due to mixture characteristics of intraligand and ligand-to-ligand charge transitions [82]. Hence, the emissions from **1**–**4** might be ascribed due to characteristics of π*–n transitions, i.e., ligand-based transition, or/and ligand-to-ligand charge transfer. Also, we found that the increase in emission intensity for **1**–**4** in the single crystal at RT is larger than that of free ligands, i.e., Na_2_bdc and 4-bpdh, by more than 2–3 folds, manifesting the role of Zn(II), which acts as a bridge to enhance the rigidity of the framework.

Finally, according to the distinctly different single crystal structures, the difference in emission properties among **1**–**4** seem to be attributed to the different architectures of the associated crystal packing. We then inspected the effects of the stereochemistry of the ligand–metal complexes and the architectures of the associated crystal packing. Unlike **1** to **3**, the absorption and emission bands for **4** tend to be red-shifted relative to those of **1**, **2**, and **3**. Due to the different coordination geometry around the center Zn(II) in **1**–**4**, the distances of Zn-O and Zn-N between the ligand and Zn(II) are different. The distances of Zn-O and Zn-N in **4** are slightly shorter than those in **1**–**3**. As a result, compound **4** exhibits a lower energy emission compared with that for **1**–**3**.

## 4. Conclusions

To sum up, based on a facile one-pot synthetic routes, we demonstrate the structural versatility of the Zn(II) centre bridged by bdc^2−^ and 4-bpdh ligands under the same synthetic conditions for building up four 3D CPs, {[Zn_2_(bdc)_2_(4-bpdh)]·C_2_H_5_OH·2H_2_O}_n_ (**1**), [Zn(bdc)(4-bpdh)]_n_ (**2**), {[Zn_2_(bdc)_2_(4-bpdh)_2_]·(4-bpdh)}_n_ (**3**), and {[Zn(bdc)(4-bpdh)]·C_2_H_5_OH}_n_ (**4**). All of their 3D MOFs are unique and different, including a SP Zn(II) coordination geometry with two-fold interpenetrating cube-like 3D coordination network for **1**, an O*_h_* coordination geometry, with non-interpenetrating 3D coordination network for **2**, a TBP coordination geometry with two-fold interpenetrating rectangular-box-like 3D coordination network for **3**, and a T*_d_* coordination geometry with five-fold interpenetrating 3D diamondoid-like coordination network for **4**, respectively. The structural characteristics of CPs **1**–**4** provide a systematic study on the factors that affect the structural diversity of their 3D MOFs, including the coordination geometry of Zn(II) centre, the coordination modes of bdc^2−^, the conformational freedom of the spacer diaza moiety (–CR=N–N=CR–) between the two pyridyl groups in the 4-bpdh ligands and guest molecules. A summary and comparisons of the structural characteristics among **1**–**4** and are listed in Table 2. These results also exemplify a unique models on the construction of the 3D MOFs, and structural interpenetration. Photoluminescence study of the compounds **1**–**4** were carried out at room temperature and showed emissions due to ligand-based transition (π*–n transition), or/and ligand-to-ligand charge transfer transitions.

## Figures and Tables

**Table 1:** Crystal data and refinement details of compounds **1**–**4**.

Compound	1	2	3	4
empirical formula	C_32_H_22_N_4_O_11_Zn_2_	C_22_H_18_N_4_O_4_Zn_1_	C_51_H_43.5_N_10_O_8_Zn_2_	C_26_H_30_N_4_O_6_Zn_1_
formula mass (g mol^−1^)	769.28	467.77	1055.20	559.91
crystal system	Triclinic	Monoclinic	Triclinic	Monoclinic
space group	*P*-1	*P*2_1_/*c*	*P*-1	*C*2/*c*
*a* (Å)	10.893(2)	7.4958(5)	10.4787(5)	27.020(2)
*b* (Å)	10.899(2)	13.5492(9)	14.7264(7)	6.0013(4)
*c* (Å)	18.088(3)	19.512(1)	15.6046(7)	20.136(1)
α (deg)	83.619(3)	90.00	98.494(1)	90.00
β (deg)	81.716(3)	90.927(2)	92.658(1)	128.095(1)
γ (deg)	88.729(3)	90.00	104.9995(9)	90.00
*V* (Å^3^)	2112.0(5)	1981.4(2)	2291.4(2)	2569.7(3)
Z	2	4	2	4
T (K)	150(2)	150(2)	200(2)	150(2)
*D*_calcd_ (g cm^−3^)	1.210	1.568	1.529	1.447
μ (mm^−1^)	1.187	1.278	1.116	1.004
θ range (deg)	1.88–25.0	1.83–27.5	1.32–27.5	1.92–27.5
total no. of data collected	22,177	18,016	29,841	9404
no. of unique data	7422	4561	10,488	2939
no. of obsd data (I > 2σ(I))	5511	3594	8875	2588
*R_int_*	0.0884	0.0588	0.0398	0.0355
refine params	456	282	612	181
*R*_1_, *wR*_2_^1^ (I > 2σ(I))	0.1073, 0.2718	0.0446, 0.1025	0.0477, 0.1307	0.0588, 0.1620
*R*_1_, *wR*_2_^1^ (all data)	0.1367, 0.2881	0.0621, 0.1103	0.0577, 0.1414	0.0653, 0.1684
GOF ^2^	1.153	1.018	1.070	1.053

^1^ *R*_1_ = ∑||*F*_o_ − *F*_c_||/∑|*F*_o_|; *wR*_2_(*F*^2^) = [∑w|*F*_o_^2^ − *F*_c_^2^|^2^/∑w(*F*_o_^4^)]^1/2^; ^2^ GOF = {∑[w|*F*_o_^2^ − *F*_c_^2^|^2^]/(*n* − *p*)}^1/2^.

**Table 2:** Summary of structural characteristics in compounds **1**–**4**.

Compound	1	2	3	4
C. N. of Zn(II)	5	6	5	4
Geometry of Zn(II) center	SP	O*_h_*	TBP	T*_d_*
Coordination mode of bdc^2−^	*bis*-bidentate	chelating/bidentate	*bis*-bidentate & *bis*-monodentate	*bis*-monodentate
Interpenetration	Two-fold	None	Two-fold	Five-fold
Guest molecule	EtOH, H_2_O	None	4-bpdh	EtOH

## Supplementary Materials

The following are available online at http://www.mdpi.com/2073-4360/9/12/644/s1, Figure S1: (a) Thermogravimetric (TG) measurement of **1**. (b) Temperature-dependent powder X-ray diffraction patterns of **1** from room temperature to 410 °C and its simulation from single-crystal diffraction data, Figure S2: (a) Thermogravimetric (TG) measurement of **2**. (b) Temperature-dependent powder X-ray diffraction patterns of **2** from room temperature to 380 °C and its simulation from single-crystal diffraction data, Figure S3: (a) Thermogravimetric (TG) measurement of **3**. (b) Temperature-dependent powder X-ray diffraction patterns of **3** from room temperature to 380 °C and its simulation from single-crystal diffraction data, Figure S4: (a) Thermogravimetric (TG) measurement of **4**. (b) Temperature-dependent powder X-ray diffraction patterns of **4** from room temperature to 380 °C and its simulation from single-crystal diffraction data, Figure S5: (a) N_2_ Gas ad-/de-sorption isotherms (b) H_2_ Gas ad-/de-sorption isotherms (c) CO_2_ Gas ad-/de-sorption isotherms for **1**–**4**, Figure S6: The UV–vis diffusive reflectance and emission spectra of 4-bpdh. λ_ex_ = 325 nm, Table S1: The yields of compounds **1**–**4** in the EtOH:H_2_O solution with different EtOH:H_2_O mixing volume ratios, Table S2: Bond lengths (Å) around Zn(II) ions in **1**, Table S3: Bond lengths (Å) around Zn(II) ions in **2**, Table S4: Bond lengths (Å) around Zn(II) ions in **3**, Table S5: Bond lengths (Å) around Zn(II) ions in **4**.
