# Supplementary material for: Four Mixed-Ligand Zn(II) Three-Dimensional Metal-Organic Frameworks: Synthesis, Structural Diversity, and Photoluminescent Property
Source: Polymers (Basel). 2017 Nov 25;9(12):644. doi: 10.3390/polym9120644 (PMC6418879; doi:10.3390/polym9120644)
Supplement: Supplementary file 1 [file polymers-09-00644-s001.pdf]

Supplementary Information

Four three-dimensional Zn(II) Mixed-ligand Metal-Organic Frameworks: Synthesis, Structural Diversity, and Photoluminescent Property

Supporting Information

Table S1. The yields of compounds **1–4** in the EtOH:H_2O solution with different EtOH:H_2O mixing volume ratios.

Table S2. Bond lengths (Å) around Zn(II) ions in **1**.

Table S3. Bond lengths (Å) around Zn(II) ions in **2**.

Table S4. Bond lengths (Å) around Zn(II) ions in **3**.

Table S5. Bond lengths (Å) around Zn(II) ions in **4**.

Figure S1 (a) Thermogravimetric (TG) measurement of **1**. (b) Temperature-dependent powder X-ray diffraction patterns of **1** from room temperature to 410 °C and its simulation from single-crystal diffraction data.

Figure S2 (a) Thermogravimetric (TG) measurement of **2**. (b) Temperature-dependent powder X-ray diffraction patterns of **2** from room temperature to 380 °C and its simulation from single-crystal diffraction data.

Figure S3 (a) Thermogravimetric (TG) measurement of **3**. (b) Temperature-dependent powder X-ray diffraction patterns of **3** from room temperature to 380 °C and its simulation from single-crystal diffraction data.

Figure S4 (a) Thermogravimetric (TG) measurement of **4**. (b) Temperature-dependent powder

X-ray diffraction patterns of **4** from room temperature to 380 °C and its simulation from single-crystal diffraction data.

Figure S5 (a) N_2 Gas ad-/de-sorption isotherms (b) H_2 Gas ad-/de-sorption isotherms (c) CO_2 Gas ad-/de-sorption isotherms for **1–4**.

Figure S6 The UV-vis diffusive reflectance and emission spectra of 4-bpdh. λ_{ex} = 325 nm.

Table S1. The yields of compounds **1–4** in the solution with different EtOH:H_2O mixing volume ratios.

EtOH:H_2O volume ratio	**Yield of 1**	**Yield of 2**	**Yield of 3**	**Yield of 4**
5:1	20.6%	23.8%		
4:1	19.7%	27.7%		
3:1	25.6%	21.7%		
2:1	28.6%	29.7%		
1:1	35.3%	24.9%		
1:2		20.0%	13.5%	28.9%
1:3		22.8%	12.2%	30.2%
1:4		18.6%	10.0%	28.6%
1:5		19.6%	4.6%	27.7%

Table S2. Bond lengths (Å) around Zn(II) ions in **1**[a]

Zn(1)–O(5)	2.011(7)	Zn(1)–O(7)$_{i}$	2.015(7)
Zn(1)–O(1)	2.028(7)	Zn(1)–N(1)	2.031(8)
Zn(1)–O(3)$_{ii}$	2.050(7)		
Zn(2)–O(6)	2.010(7)	Zn(2)–N(4)$_{iii}$	2.012(8)
Zn(2)–O(2)	2.018(8)	Zn(2)–O(4)$_{ii}$	2.024(8)
Zn(2)–O(8)$_{i}$	2.025(7)		
O(5)–Zn(1)–O(7)$_{i}$	160.0(3)	O(5)–Zn(1)–O(1)	88.2(4)
O(7)$_{i}$–Zn(1)–O(1)	89.1(4)	O(5)–Zn(1)–N(1)	99.6(3)
O(7)$_{i}$–Zn(1)–N(1)	100.3(3)	O(1)–Zn(1)–N(1)	103.7(3)
O(5)–Zn(1)–O(3)$_{ii}$	87.8(4)	O(7)$_{i}$–Zn(1)–O(3)$_{ii}$	86.9(4)
O(1)–Zn(1)–O(3)$_{ii}$	156.8(3)	N(1)–Zn(1)–O(3)$_{ii}$	103.7(3)
O(6)–Zn(2)–N(4)$_{iii}$	100.9(3)	O(6)–Zn(2)–O(2)	88.8(4)
N(4)$_{iii}$–Zn(2)–O(2)	97.1(4)	O(6)–Zn(2)–O(4)$_{ii}$	87.6(4)
N(4)$_{iii}$–Zn(2)– O(4)$_{ii}$	101.5(4)	O(2)–Zn(2)–O(4)$_{ii}$	161.4(3)
O(6)–Zn(2)–O(8)$_{i}$	159.6(3)	N(4)$_{iii}$–Zn(2)–O(8)$_{i}$	99.5(3)
O(2)–Zn(2)–O(8)$_{i}$	88.7(4)	O(4)$_{ii}$–Zn(2)–O(8)$_{i}$	88.4(4)

[a]Symmetry transformations used to generate equivalent atoms : i = x+1, y, z; ii = x, y–1, z; iii = x, y, z+1.

Table S3. Bond lengths (Å) around Zn(II) ions in **2**[a]

Zn(1)–O(2)$_{i}$	2.045(2)	Zn(1)–O(1)	2.051(2)
Zn(1)–O(4)$_{ii}$	2.090(2)	Zn(1)–N(1)	2.155(2)
Zn(1)–N(4)	2.226(2)	Zn(1)–O(3)$_{ii}$	2.354(3)
O(2)$_{i}$–Zn(1)–O(1)	100.20(7)	O(2)$_{i}$–Zn(1)–O(4)$_{ii}$	162.5(8)
O(1)–Zn(1)–O(4)$_{ii}$	92.35(8)	O(2)$_{i}$–Zn(1)–N(1)	93.16(8)
O(1)–Zn(1)–N(1)	104.48(8)	O(4)$_{ii}$–Zn(1)–N(1)	95.49(8)
O(2)$_{i}$–Zn(1)–N(4)	83.07(8)	O(1)–Zn(1)–N(4)	87.51(8)
O(4)$_{ii}$–Zn(1)–N(4)	85.34(8)	N(1)–Zn(1)–N(4)	167.91(9)
O(2)$_{i}$–Zn(1)–O(3)$_{ii}$	106.07(7)	O(1)–Zn(1)–O(3)$_{ii}$	150.04(7)
O(4)$_{ii}$–Zn(1)–O(3)$_{ii}$	59.10(7)	N(1)–Zn(1)–O(3)$_{ii}$	88.37(8)
N(4)–Zn(1)–O(3)$_{ii}$	81.70(8)		

[a]Symmetry transformations used to generate equivalent atoms : i = –x, –y, –z; ii = x, –y+1/2, z+1/2.

Table S4. Bond lengths (Å) around Zn(II) ions in **3**[a]

Zn(1)–O(5)	1.972(2)	Zn(1)–O(3)	2.020(2)
Zn(1)–O(1)	2.034(2)	Zn(1)–N(1)	2.141(2)
Zn(1)–N(4)	2.228(3)		
Zn(2)–O(7)	1.964(2)	Zn(2)–O(4)	1.979(2)
Zn(2)–O(2)	2.001(2)	Zn(2)–N(5)	2.178(2)
Zn(2)–N(8)	2.216(3)		
O(5)–Zn(1)–O(3)	108.77(9)	O(5)–Zn(1)–O(1)	119.9(1)
O(3)–Zn(1)–O(1)	130.04(9)	O(5)–Zn(1)–N(1)	101.9(1)
O(3)–Zn(1)–N(1)	90.21(9)	O(1)–Zn(1)–N(1)	90.43(9)
O(5)–Zn(1)–N(4)	85.6(1)	O(3)–Zn(1)–N(4)	82.26(9)
O(1)–Zn(1)–N(4)	86.70(9)	N(1)–Zn(1)–N(4)	172.4(1)
O(7)–Zn(2)–O(4)	110.3(1)	O(7)–Zn(2)–O(2)	117.71(9)
O(4)–Zn(2)–O(2)	131.3(1)	O(7)–Zn(2)–N(5)	97.4(1)
O(4)–Zn(2)–N(5)	93.1(1)	O(2)–Zn(2)–N(5)	88.53(9)
O(7)–Zn(2)–N(8)	85.2(1)	O(4)–Zn(2)–N(8)	90.1(1)
O(2)–Zn(2)–N(8)	86.38(9)	N(5)–Zn(2)–N(8)	174.9(1)

Table S5. Bond lengths (Å) around Zn(II) ions in **4**[a]

Zn(1)–O(1)	1.936(2)	Zn(1)–O(1)$_i$	1.936(2)
Zn(1)–N(1)	2.032(2)	Zn(1)–N(1)$_i$	2.032(2)
O(1)–Zn–O(1)$_i$	104.3(1)	O(1)–Zn–N(1)	122.9(1)
O(1)$_i$–Zn–N(1)	102.0(1)	O(1) –Zn–N(1)$_i$	102.0(1)
O(1)$_i$–Zn–N(1)$_i$	122.9(1)	N(1)–Zn–N(1)$_i$	104.4(2)

[a]Symmetry transformations used to generate equivalent atoms : i = –x+1, y, –z+1/2.

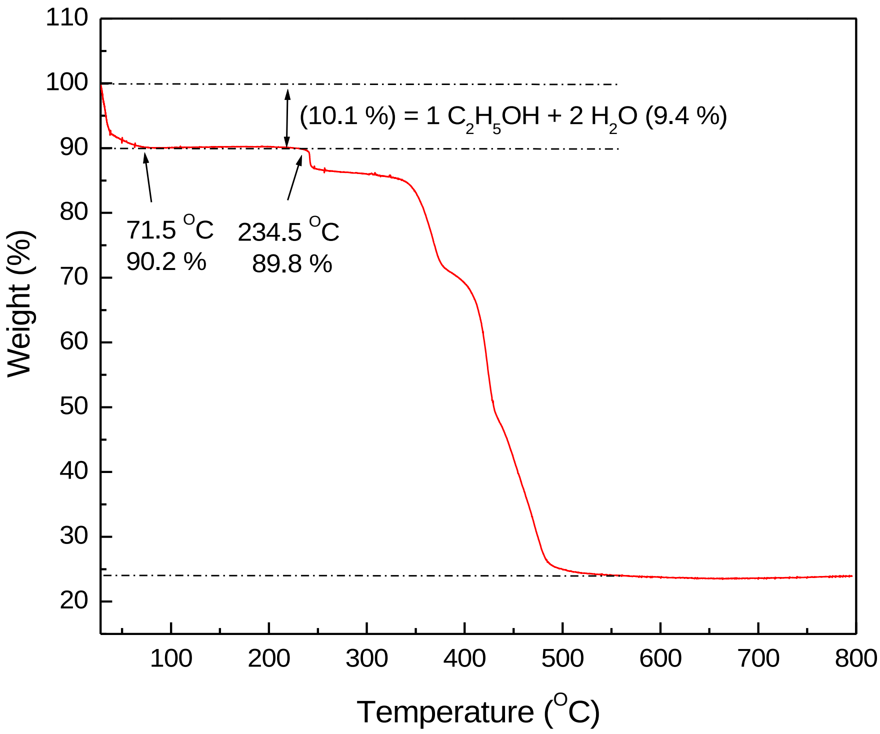

(a)

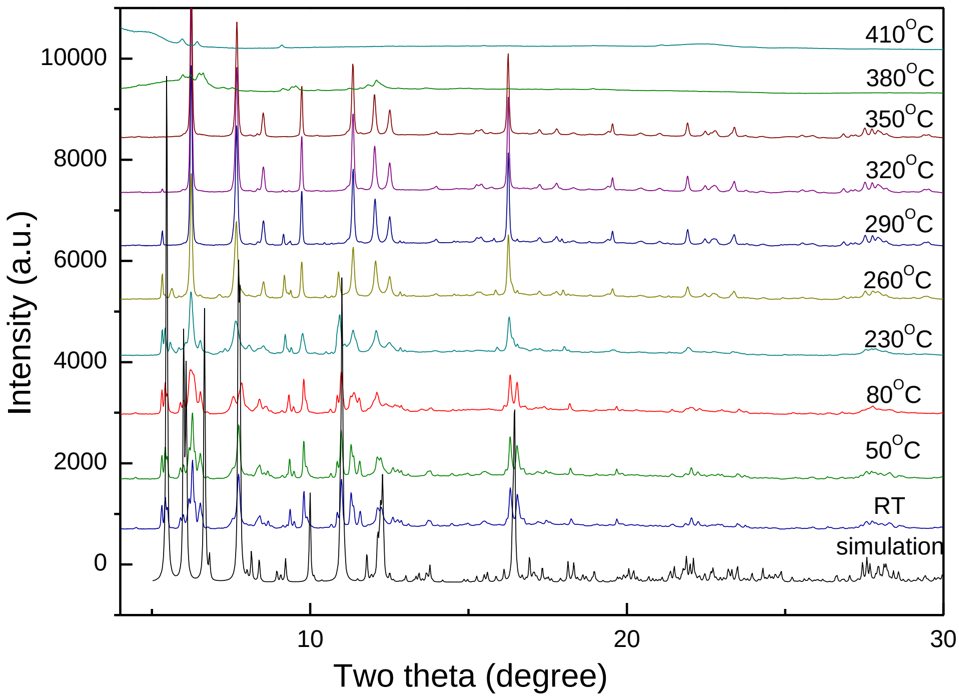

(b)

Figure S1 (a) Thermogravimetric (TG) measurement of **1**. (b) Temperature-dependent in-situ powder X-ray diffraction patterns of **1** from room temperature to 410 °C and its simulation from single-crystal diffraction data.

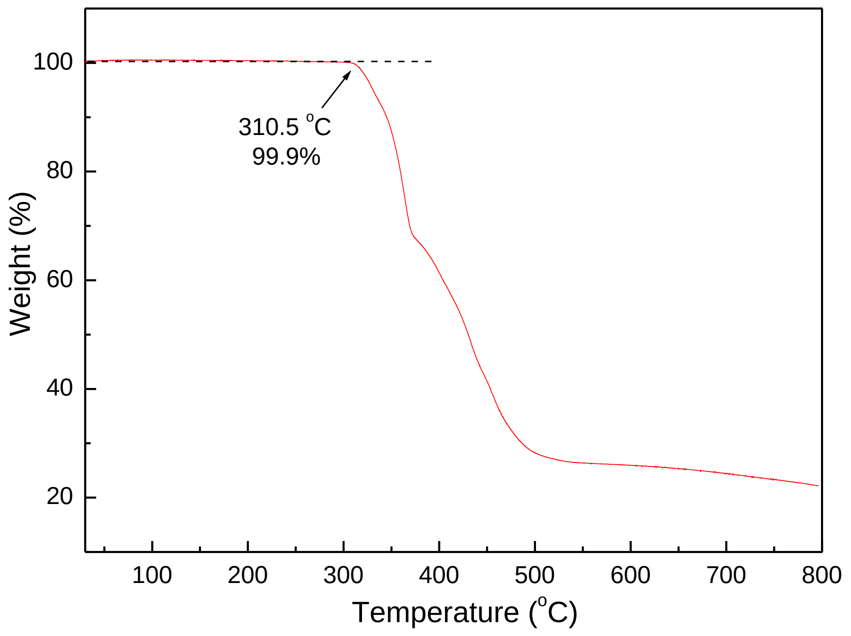

(a)

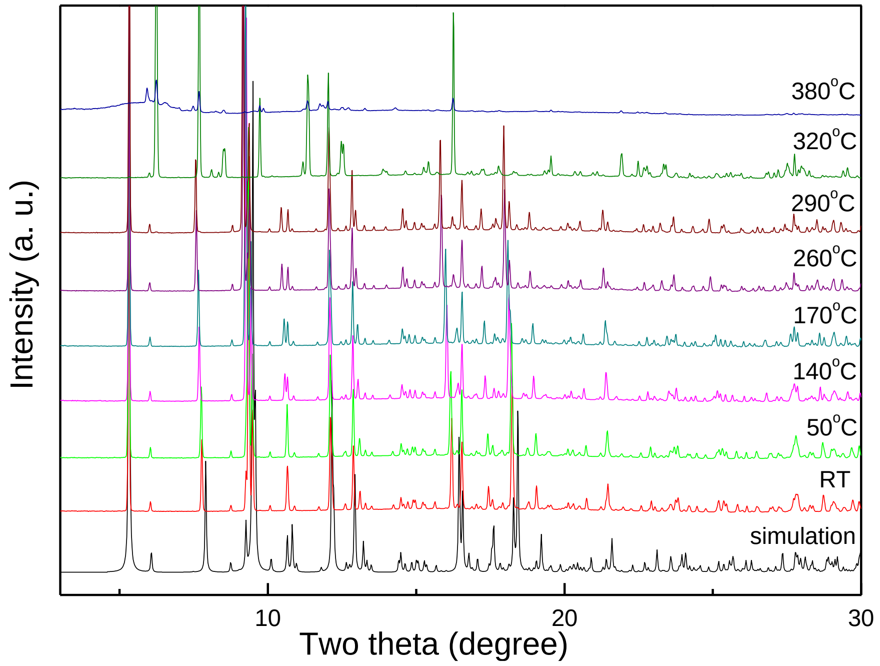

(b)

Figure S2 (a) Thermogravimetric (TG) measurement of **2**. (b) Temperature-dependent in-situ powder X-ray diffraction patterns of **2** from room temperature to 380 °C and its simulation from single-crystal diffraction data.

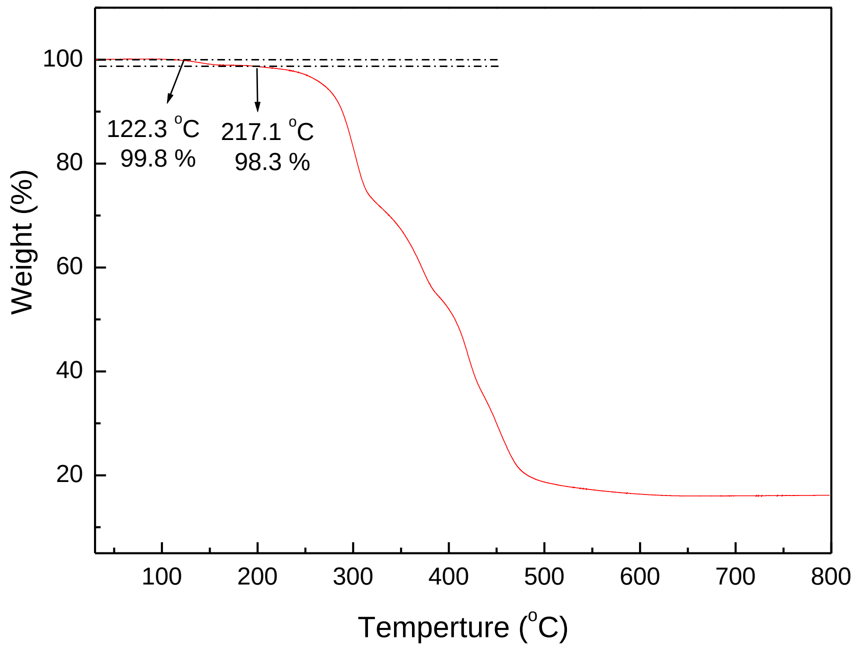

(a)

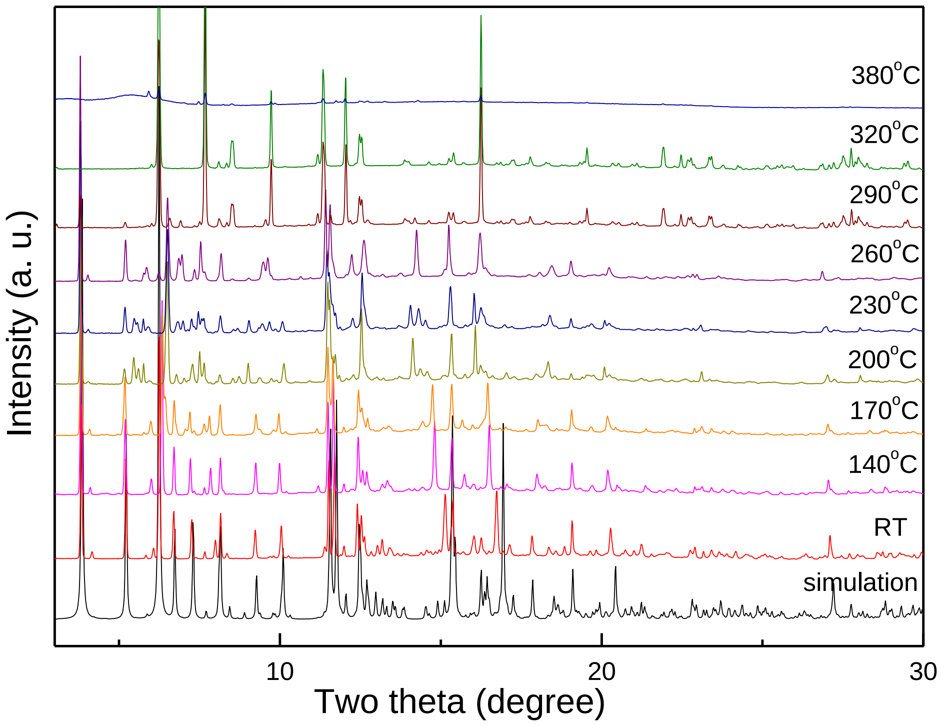

(b)

Figure S3 (a) Thermogravimetric (TG) measurement of **3**. (b) Temperature-dependent in-situ powder X-ray diffraction patterns of **3** from room temperature to 380 °C and its simulation from single-crystal diffraction data.

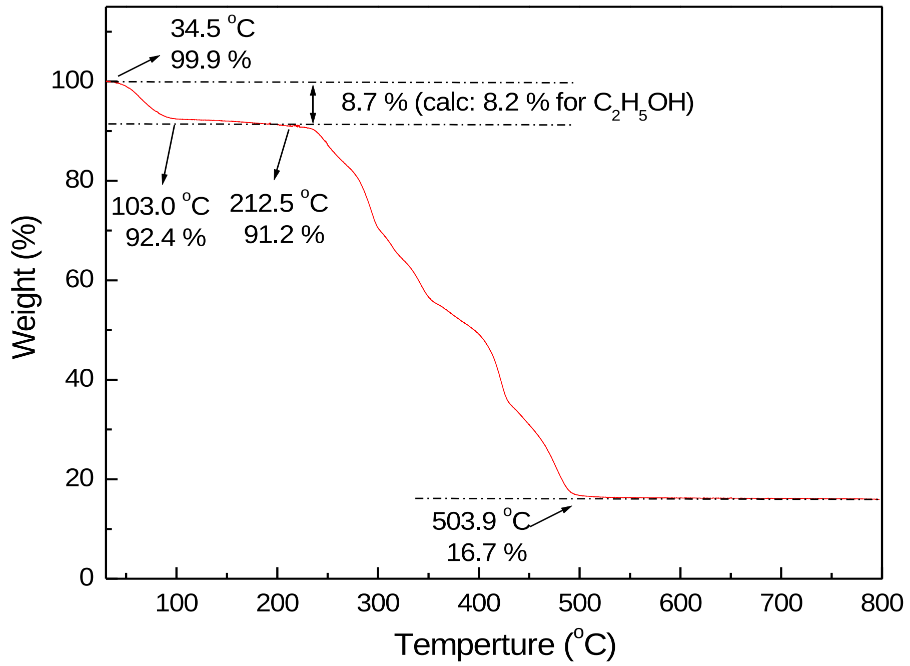

(a)

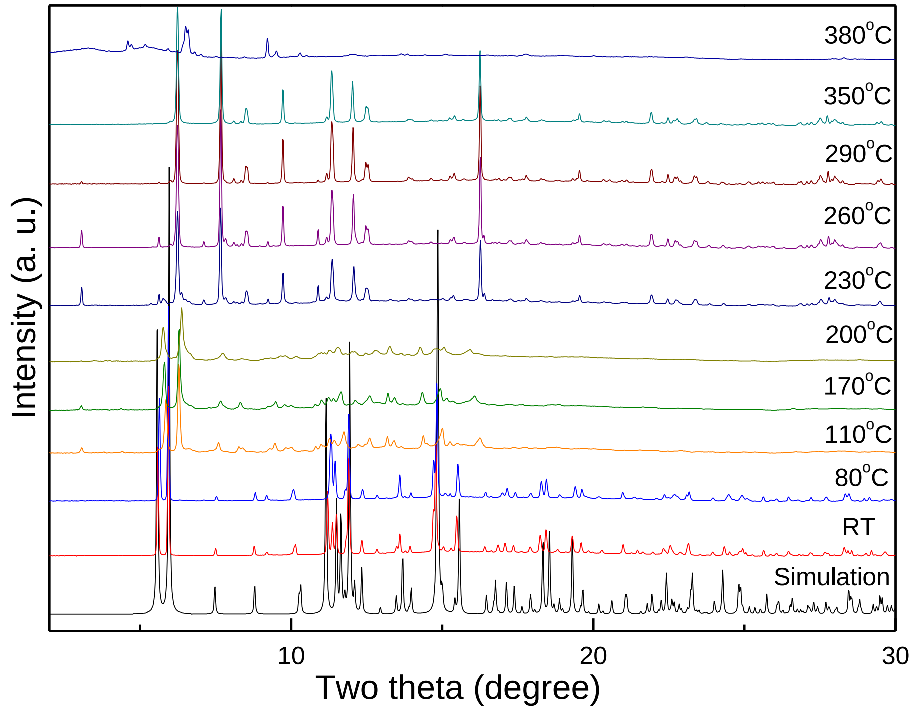

(b)

Figure S4 (a) Thermogravimetric (TG) measurement of **4**. (b) Temperature-dependent in-situ powder X-ray diffraction patterns of **4** from room temperature to 380 °C and its simulation from single-crystal diffraction data.

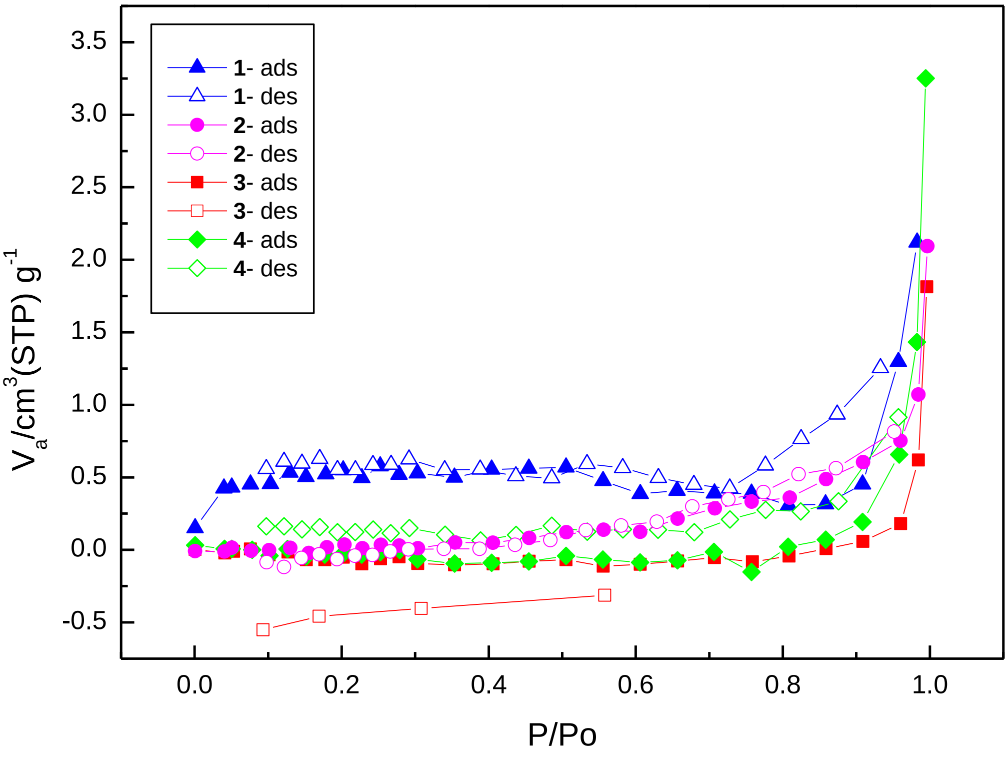

1- ads
1- des
2- ads
2- des
3- ads
3- des
4- ads
4- des
V_a/cm^3(STP) g^-1
P/Po
3.5
3.0
2.5
2.0
1.5
1.0
0.5
0.0
-0.5
0.0
0.2
0.4
0.6
0.8
1.0

(a)

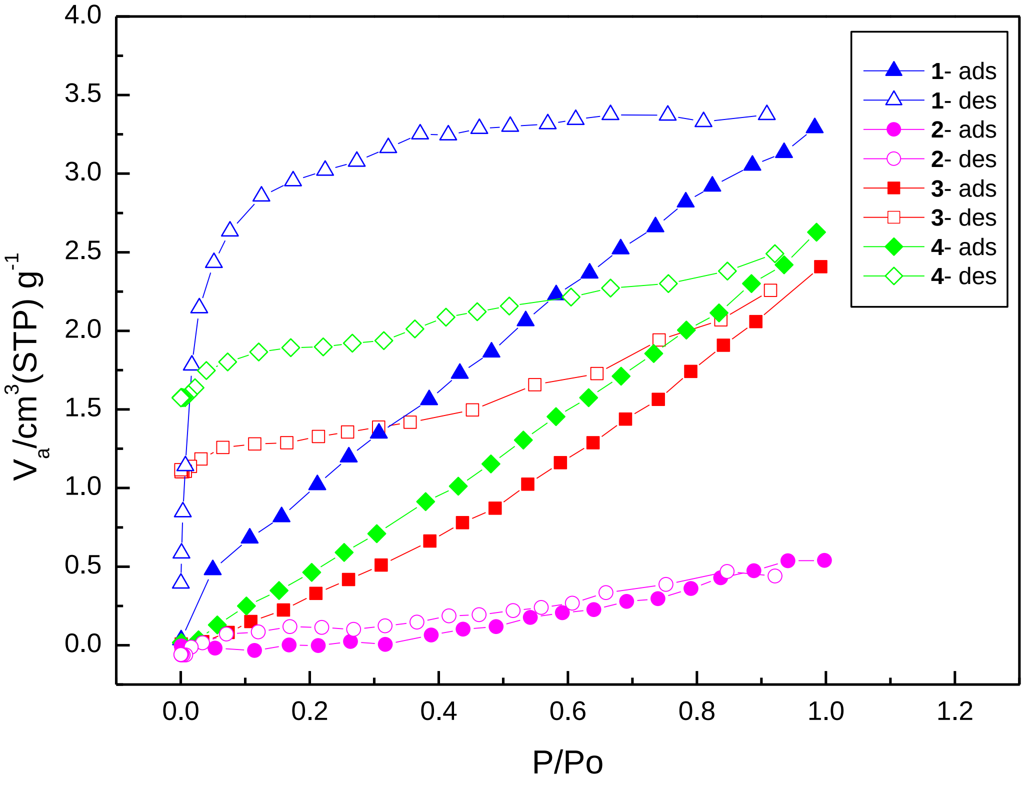

1- ads
1- des
2- ads
2- des
3- ads
3- des
4- ads
4- des
V_a/cm^3(STP) g^-1
P/Po
4.0
3.5
3.0
2.5
2.0
1.5
1.0
0.5
0.0
0.0
0.2
0.4
0.6
0.8
1.0
1.2

(b)

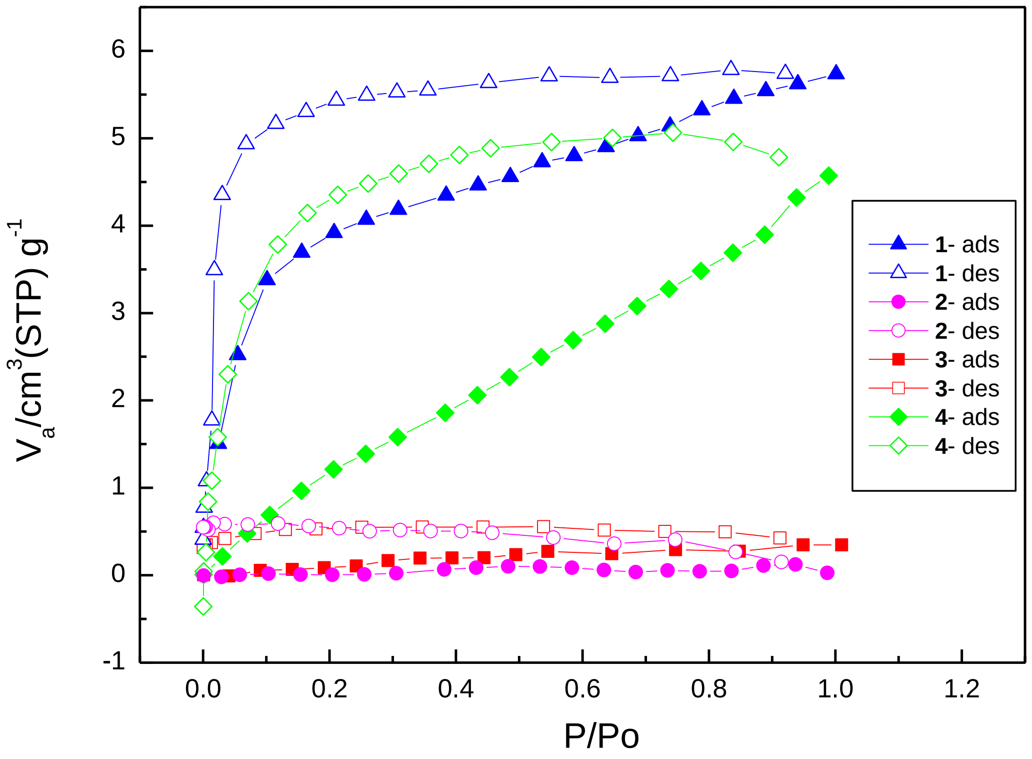

(c)

Figure S5 (a) N_2 Gas ad-/de-sorption isotherms (b) H_2 Gas ad-/de-sorption isotherms (c) CO_2 Gas ad-/de-sorption isotherms for **1–4**.

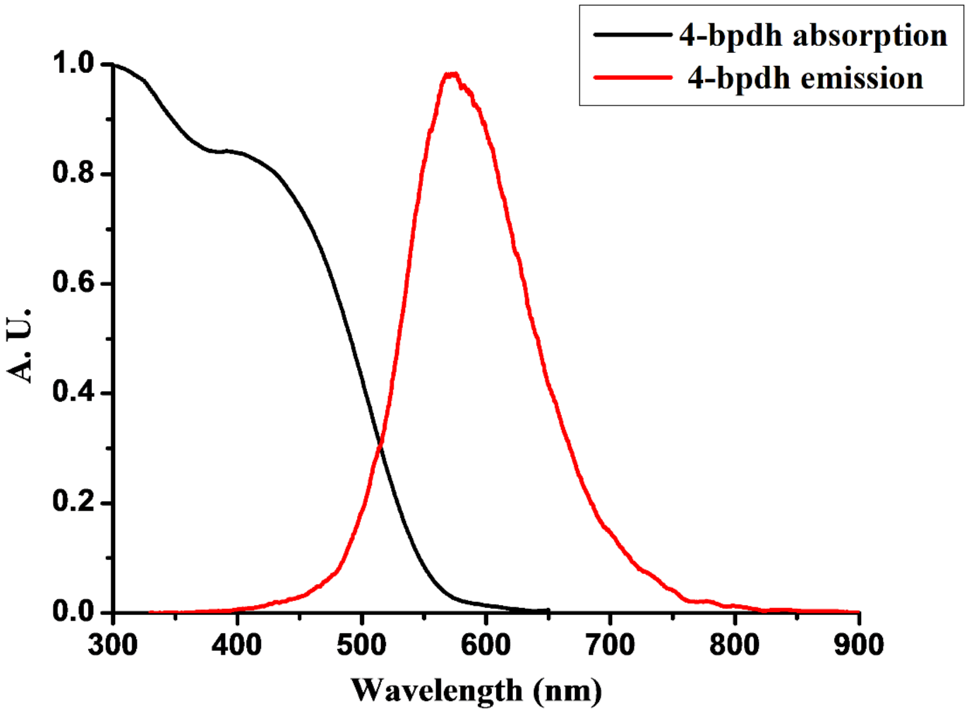

Fig. S6 The UV-vis diffusive reflectance and emission spectra of 4-bpdh. λ_{ex} = 325 nm.
